# Distribution and kinetics of the Kv1.3-blocking peptide HsTX1[R14A] in experimental rats

**DOI:** 10.1038/s41598-017-03998-x

**Published:** 2017-06-16

**Authors:** Ralf Bergmann, Manja Kubeil, Kristof Zarschler, Sandeep Chhabra, Rajeev B. Tajhya, Christine Beeton, Michael W. Pennington, Michael Bachmann, Raymond S. Norton, Holger Stephan

**Affiliations:** 1 Helmholtz-Zentrum Dresden - Rossendorf, Institute of Radiopharmaceutical Cancer Research, Dresden, D-01328 Germany; 2 School of Chemistry, Monash University, Melbourne, Victoria 3800 Australia; 3 Medicinal Chemistry, Monash Institute of Pharmaceutical Sciences, Monash University, Parkville, Victoria 3052 Australia; 4 Department of Molecular Physiology and Biophysics, Baylor College of Medicine, Houston, TX 77030 USA; 5 Peptides International, Louisville, KY 40299 USA

## Abstract

The peptide HsTX1[R14A] is a potent and selective blocker of the voltage-gated potassium channel Kv1.3, which is a highly promising target for the treatment of autoimmune diseases and other conditions. In order to assess the biodistribution of this peptide, it was conjugated with NOTA and radiolabelled with copper-64. [^64^Cu]Cu-NOTA-HsTX1[R14A] was synthesised in high radiochemical purity and yield. The radiotracer was evaluated *in vitro* and *in vivo*. The biodistribution and PET studies after intravenous and subcutaneous injections showed similar patterns and kinetics. The hydrophilic peptide was rapidly distributed, showed low accumulation in most of the organs and tissues, and demonstrated high molecular stability *in vitro* and *in vivo*. The most prominent accumulation occurred in the epiphyseal plates of trabecular bones. The high stability and bioavailability, low normal-tissue uptake of [^64^Cu]Cu-NOTA-HsTX1[R14A], and accumulation in regions of up-regulated Kv channels both *in vitro* and *in vivo* demonstrate that HsTX1[R14A] represents a valuable lead for conditions treatable by blockade of the voltage-gated potassium channel Kv1.3. The pharmacokinetics shows that both intravenous and subcutaneous applications are viable routes for the delivery of this potent peptide.

## Introduction

Voltage-gated potassium (Kv) channels are integral membrane proteins that regulate cell membrane potential and are involved in a variety of cellular functions including apoptosis and cell volume regulation^1^. Elevated expression of the voltage-gated channel Kv1.3 in effector memory T (T_EM_) lymphocytes is implicated in the pathology of a range of autoimmune diseases, including multiple sclerosis, rheumatoid arthritis, systemic lupus erythematosus and type 1 diabetes^2^, as well as non-autoimmune conditions such as asthma, chronic obstructive pulmonary disease and graft-versus-host disease, and therefore Kv1.3 channels are a highly promising therapeutic target for the treatment of such diseases^3, 4^. The sea anemone-derived peptide ShK, from *Stichodactyla helianthus*, exhibits high affinity for these channels, with an IC_50_ value of 11 pM^5^. ShK and its analogues significantly reduce disease severity in several animal models of T_EM_ lymphocyte-related diseases including delayed-type hypersensitivity, chronic relapsing-remitting experimental autoimmune encephalomyelitis, pristane-induced arthritis and asthma via blockade of Kv1.3 channels in T_EM_ cells^6–9^. One ShK analogue, ShK-186 (dalazatide), has completed phase I human clinical trials following subcutaneous administration. It was well tolerated and achieved clinical improvement in target lesions in patients with moderate plaque psoriasis^10^. These promising preclinical and clinical studies warrant the ongoing development of Kv1.3 channel blockers for the treatment of a variety of immune-related diseases.

HsTX1 toxin, from the scorpion *Heterometrus spinnifer*, is a 34-residue, C-terminally amidated peptide cross-linked by four disulfide bridges. The native peptide blocks Kv1.3 channels with similar affinity to ShK peptide analogues^11, 12^, and an analogue, HsTX1[R14A], has been developed recently that not only retains the high affinity of the naturally-occurring peptide, but also exhibits an approximate 2000-fold greater selectivity for Kv1.3 channels over other potassium channels^13, 14^. HsTX1[R14A] therefore represents a very promising therapeutic candidate for the treatment of the above-mentioned diseases.

Although HsTX1[R14A] adopts a very stable structure that is resistant to proteolysis^14^, an oral route of administration appears unlikely. We have therefore explored buccal^15–17^ and pulmonary^18^ delivery of this peptide, both of which proved to be effective. We are also exploring slow-release formulations (unpublished). An important question that arises in considering the optimal route of administration and frequency of dosing, however, is the lifetime of the peptide *in vivo* and its tissue distribution. In the case of ShK-186, for example, a ^111^In-labelled 1,4,7,10-tetraazacyclododecane-1,4,7,10-tetraacetic acid conjugate was used to assess whole-blood pharmacokinetic parameters as well as peptide absorption, distribution, and excretion. ShK-186 was absorbed slowly from the injection site, resulting in blood concentrations above the Kv1.3 channel-blocking IC_50_ value for up to 7 days in monkeys^8^. In delayed-type hypersensitivity, chronic relapsing-remitting experimental autoimmune encephalomyelitis, and pristane-induced arthritis rat models, a single dose of ShK-186 every 2 to 5 days was as effective as daily administration^8^. The slow dissemination of ShK-186 from the injection site and its long residence time on the Kv1.3 channel contribute to its prolonged therapeutic effect in animal models of autoimmune disease. In this study we have used HsTX1[R14A] modified at its N-terminus with a 1,4,7-triazacyclononane-triacetic acid (NOTA) tag for labelling with ^64^Cu as an ideal positron emitter^19, 20^, enabling positron emission tomography (PET) studies of peptide distribution in rats over a period of days. The results show a long *in vivo* half-life as a result of slow renal clearance of the peptide. In view of the high potency and selectivity of HsTX1[R14A] for the target channel Kv1.3, and the importance of this channel as a therapeutic target^6, 21^, the persistence of this peptide *in vivo* strengthens the case for its further development as a therapeutic for the treatment of the above-mentioned immune-related diseases.

## Results

### Peptide characterisation

**Figure 1:**
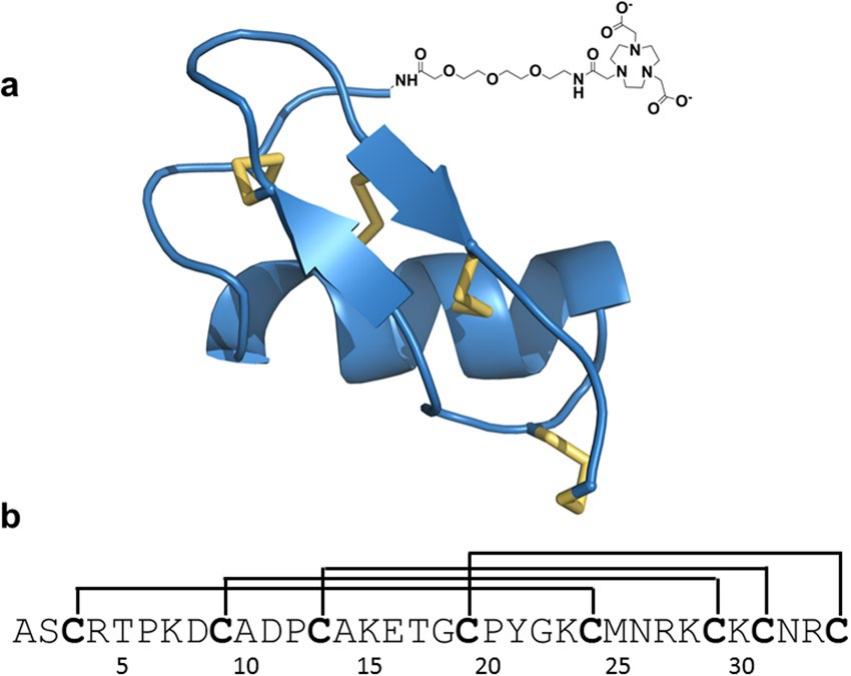
Structure of HsTX1[R14A] conjugate. (**a**) Simulated structure of HsTX1[R14A]^14^ with the NOTA tag at the N-terminus shown schematically. (**b**) Amino acid sequence of HsTX1[R14A] showing the positions of the four disulfide bridges^11^.

HsTX1[R14A] was synthesised as described previously^14^ but with a NOTA tag coupled to the N-terminus via an aminoethyloxyethyloxyacetyl (AeeA) linker, as shown in Fig. 1. NOTA-HsTX1[R14A] folded rapidly to a single major product, resulting in the typical pattern of a major earlier-eluting peak by RP-HPLC followed by later-eluting misfolded species and side-products (Supplementary Fig. S1). When tested against the voltage-gated potassium channel Kv1.3 expressed in L929 mouse fibroblast cells, the tagged peptide had an IC_50_ of 68 ± 12 pM (Supplementary Fig. S2), which was close enough to that of HsTX1[R14A] (IC_50_ 45 ± 3 pM) to confirm that the tagged peptide was an excellent mimic of the parent peptide for the purpose of this study.

### Radiolabelling

The peptide HsTX1[R14A] with a NOTA tag was efficiently labelled with ^64^Cu^II^ (half-life 12.7 h). The ^64^Cu^II^ complex was formed in a concentration range of 5 to 100 µg/100 µL NOTA-peptide conjugate within 30 min at 37 °C, employing 35–55 MBq (0.95–1.5 mCi) of [^64^Cu]CuCl_2_. Radio-TLC and radio-HPLC exhibit a single peak of [^64^Cu]Cu-NOTA-HsTX1[R14A] and no trace of free ^64^Cu^II^ (Supplementary Figs S3 and S4). The ^64^Cu^II^ complex formed was stable in the presence of 0.1 M aqueous EDTA solution for at least 24 h. These results verify that NOTA is an appropriate bifunctional chelating agent for ^64^Cu-labelling of peptides^22, 23^, and the corresponding ^64^Cu-labelled peptide HsTX1[R14A] can be utilised to obtain reliable information about the biodistribution.

### Biodistribution

**Figure 2:**
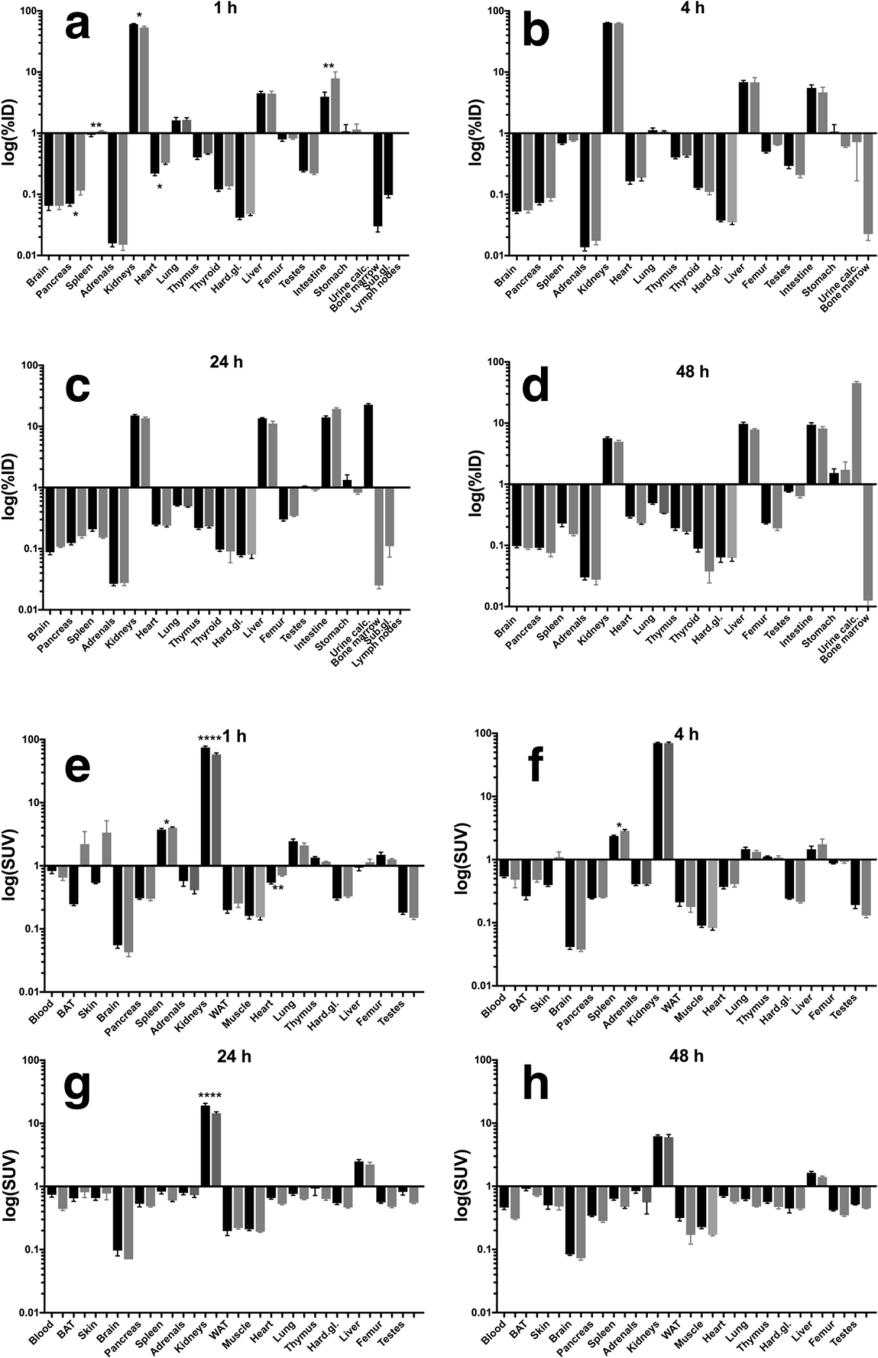
Biodistribution of the [^64^Cu]Cu-NOTA-HsTX1[R14A] in rats after single injection (i.v., black bars, n = 12; s.c., gray bars, n = 4). The data are presented as %ID (**a–d**) or SUV (**e**–**h**) in means ± SEM; BAT – brown adipose tissue, WAT – white adipose tissue, Hard. Gl. – Harderian glands, Sub. gl. – submandibular glands, lymph nodes – submandibular lymph nodes, urine calc. – urine calculated as difference between injected dose and recovery.

[^64^Cu]Cu-NOTA-HsTX1[R14A] peptide biodistribution was studied by organ and tissue extraction (Fig. 2; Supplementary Tables S1 and S2) and by small animal PET. [^64^Cu]Cu-NOTA-HsTX1[R14A] was injected intravenously (i.v.) to study the biodistribution and kinetics after direct injection into the circulation. A subcutaneous (s.c.) injection was carried out to evaluate the distribution and kinetics of the radiotracer with first contact to the lymphatic system and in direct comparison to i.v. administration.

As a relatively low molecular mass peptide (4164 Da before complexation with Cu), the [^64^Cu]Cu-NOTA-HsTX1[R14A] was rapidly distributed in the circulation after i.v. injection. The s.c. injection caused a delay in the ^64^Cu activity accumulation in several tissues. After 60 min this effect was significant in the spleen, pancreas, kidneys, heart and the remaining body (Supplementary Table S1). However, due to the fast diffusion from the injection site into the circulation, no significant difference in the whole blood concentration between the two injection types could be detected after 1 h. After 4 h, the spleen and femur represent the only organs where the ^64^Cu activity concentration is significantly different between i.v. and s.c. injection. At later time points (1–2 days after injection), this difference had disappeared (Supplementary Tables S1 and S2).

**Figure 3:**
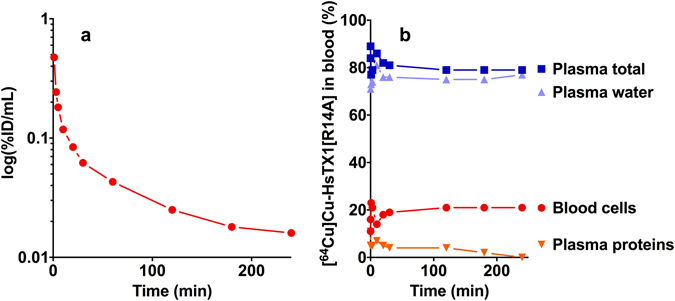
Distribution in the blood. (**a**) Representative time activity curve of the [^64^Cu]Cu-NOTA-HsTX1[R14A] after single i.v. injection in the arterial blood with metabolite correction. (**b**) Distribution of the radiotracer in the blood compartments (blood cells, plasma, plasma water, plasma proteins) of a rat. The values are expressed as % of total blood activity.

The analysis of the blood after i.v. injection (blood activity concentration after s.c. injection is too low for analysis) showed that the distribution of [^64^Cu]Cu-NOTA-HsTX1[R14A] in the different blood compartments (Fig. 3) did not change during the time of detection (0–4 h). It was located primarily in the blood plasma (82 ± 4% of the whole blood ^64^Cu activity) with 75 ± 3% in the plasma water and only 5 ± 3% bound to plasma proteins.

**Figure 4:**
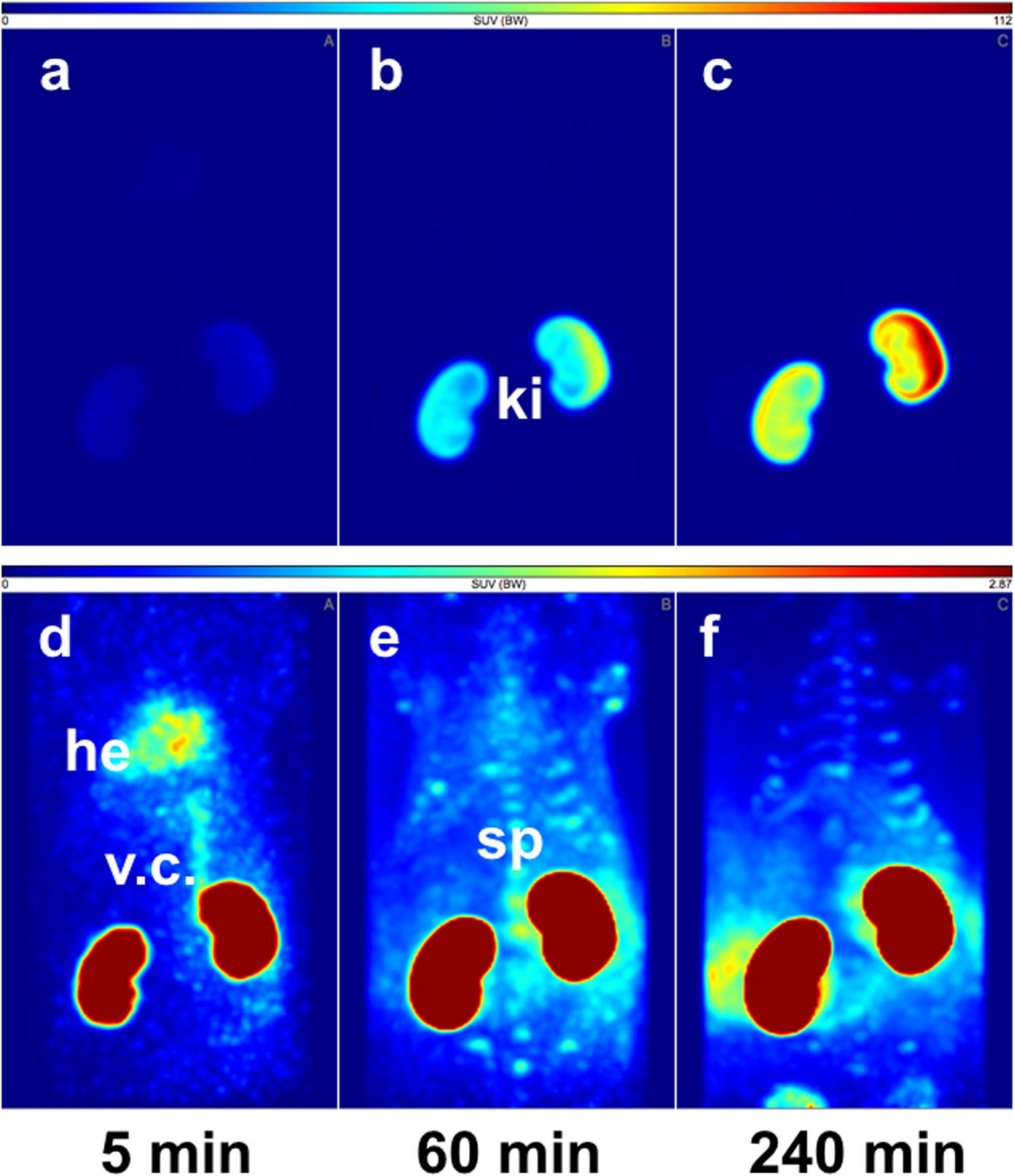
PET-biodistribution after i.v. injection. Maximum Intensity Projections (MIP) of a PET-study with [^64^Cu]Cu-NOTA-HsTX1[R14A] in a healthy Wistar rat at 5, 60 and 240 min after a single intravenous injection into a tail vein; (**a–c**) images scaled to the maximum intensity SUV 112 g/mL. (**d–f**) Images rescaled to SUV 2.87 g/mL, (ki, kidney; he, heart; v.c., vena cava; sp, spine).

**Figure 5:**
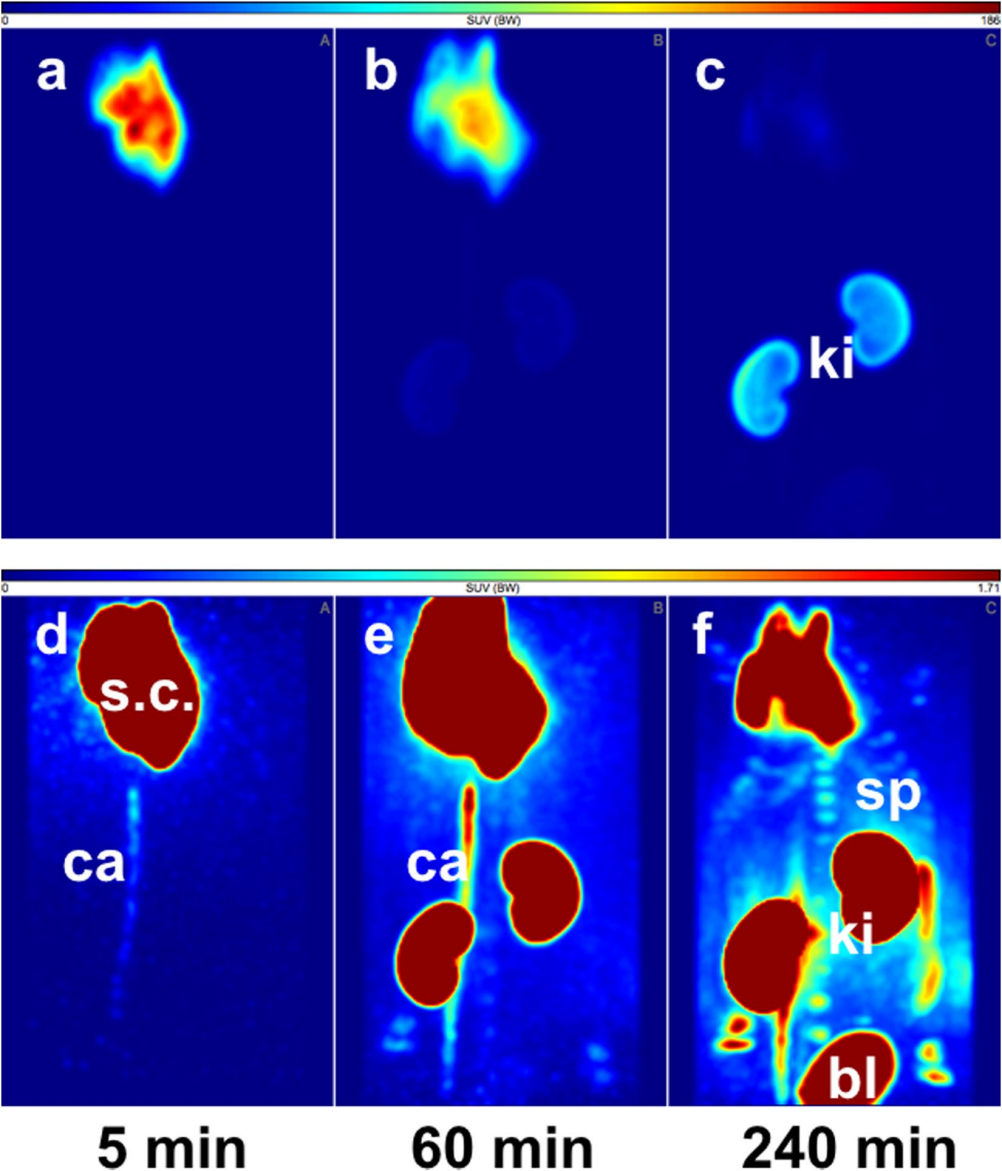
PET-biodistribution after s.c. injection. Maximum Intensity Projections (MIP) of a PET-study with [^64^Cu]Cu-NOTA-HsTX1[R14A] in a healthy Wistar rat at 5, 60 and 240 min after a single subcutaneous injection in the back; (**a–c**) images scaled to the maximum intensity SUV 186 g/mL. (**d–f** ) Images rescaled to SUV 1.71 g/mL, (ki, kidney; s.c., subcutaneous injection site; ca, catheter; sp, spine; bl, bladder).

**Figure 6:**
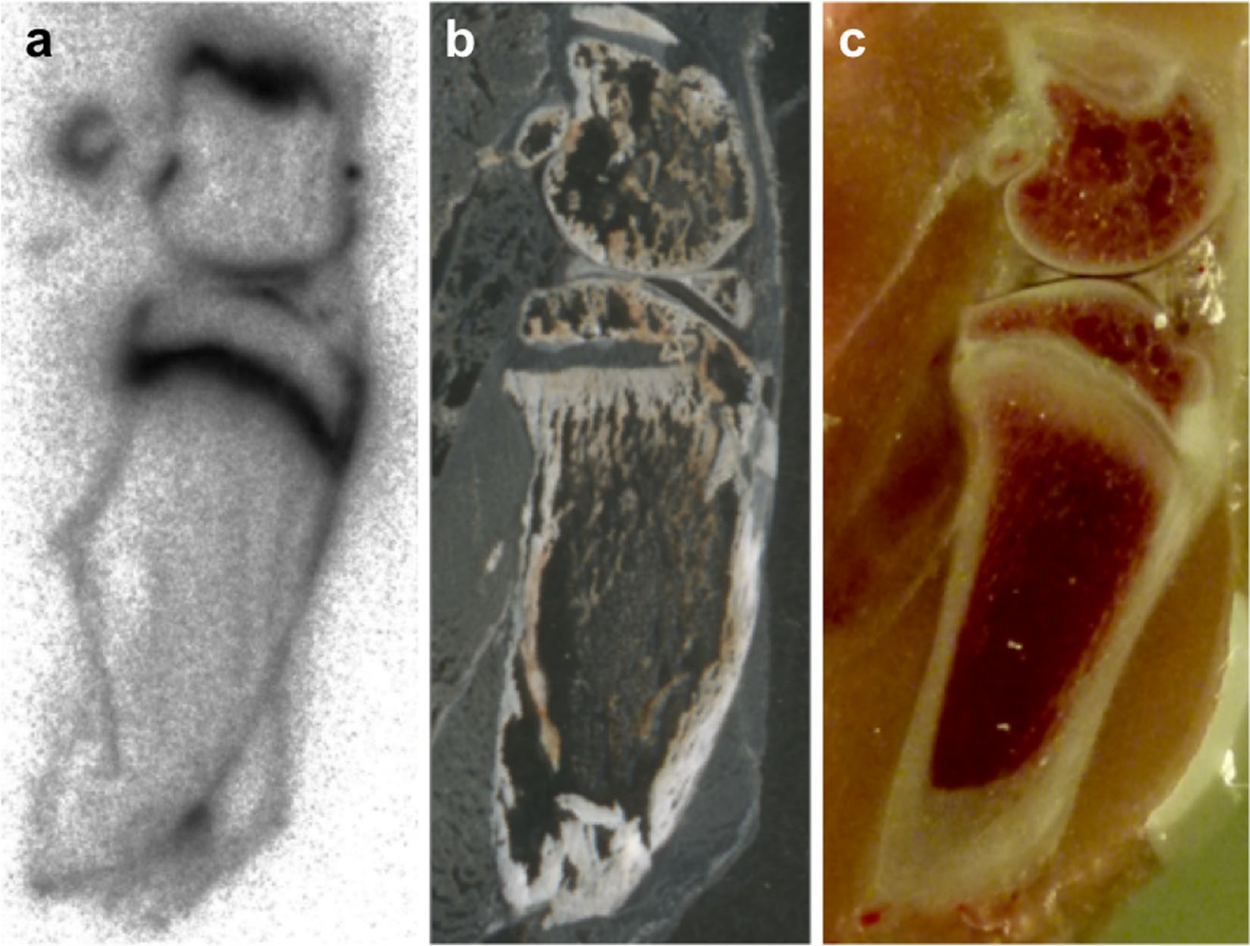
Distribution in the bone. Representative (**a**) radioluminogram, (**b**) histological frozen section (20 µm), and (**c**) photography from 4 independent studies of rat femur surface 4 h after single intravenous injection of [^64^Cu]Cu-NOTA-HsTX1[R14A].

The PET data (Figs 4 and 5) show that the biodistribution of the radiotracer after 5 h was very similar in both animals, independent of the site of injection. The peptide was mainly eliminated renally and in parallel the liver uptake was relatively low. No lymph nodes were detectable in the images. In the late frames of the PET studies (Figs 4f and 5f), but also in the biodistribution (Tables S1 and S2), activity was visible in the skeleton. In the extractive biodistribution experiments of selected organs and tissues, increased activity was detected in the femur. As this localisation of the radiotracer in the bone was unexpected, an additional study was carried out in which the femurs were extracted 4 h after injection, frozen and cut. The sections were studied by autoradiography. In Fig. 6, a typical autoradiogram, section and image of the surface of a femur are presented. The detailed autoradiographic study of the activity distribution showed that [^64^Cu]Cu-NOTA-HsTX1[R14A] was located primarily in the growth plate, the region of increased metabolism. This bone activity accumulation pattern and kinetics were, however, different from directly bone-seeking agents like ^99m^Tc-MDP or ^18^F^−^ or ^68^Ga-bisphosphonates^24, 25^.

The kinetic analysis of the dynamic standard uptake ratio (tissue to blood ratios) PET data of the kidneys, liver, and bone marrow (epiphyseal plates) showed linear tissue to blood time activity curves for the standardised uptake ratio SUR (Supplementary Fig. S5). This is most likely the result of the radiotracer accumulation in these organs and tissues according to a two-compartment model with irreversible binding, like it is typical for [^18^F]fluoro-deoxy-D-glucose accumulation in many tissues^26, 27^. The elimination organs kidney and liver trapped the radiolabelled peptide. The shape of the bone marrow SUR curve could be an indication that cells in the bone marrow bind and retain the [^64^Cu]Cu-NOTA-HsTX1[R14A] that explains the clearly visible accumulation of the radiotracer in the region of the epiphyseal plate of the bone marrow (Fig. 6).

**Figure 7:**
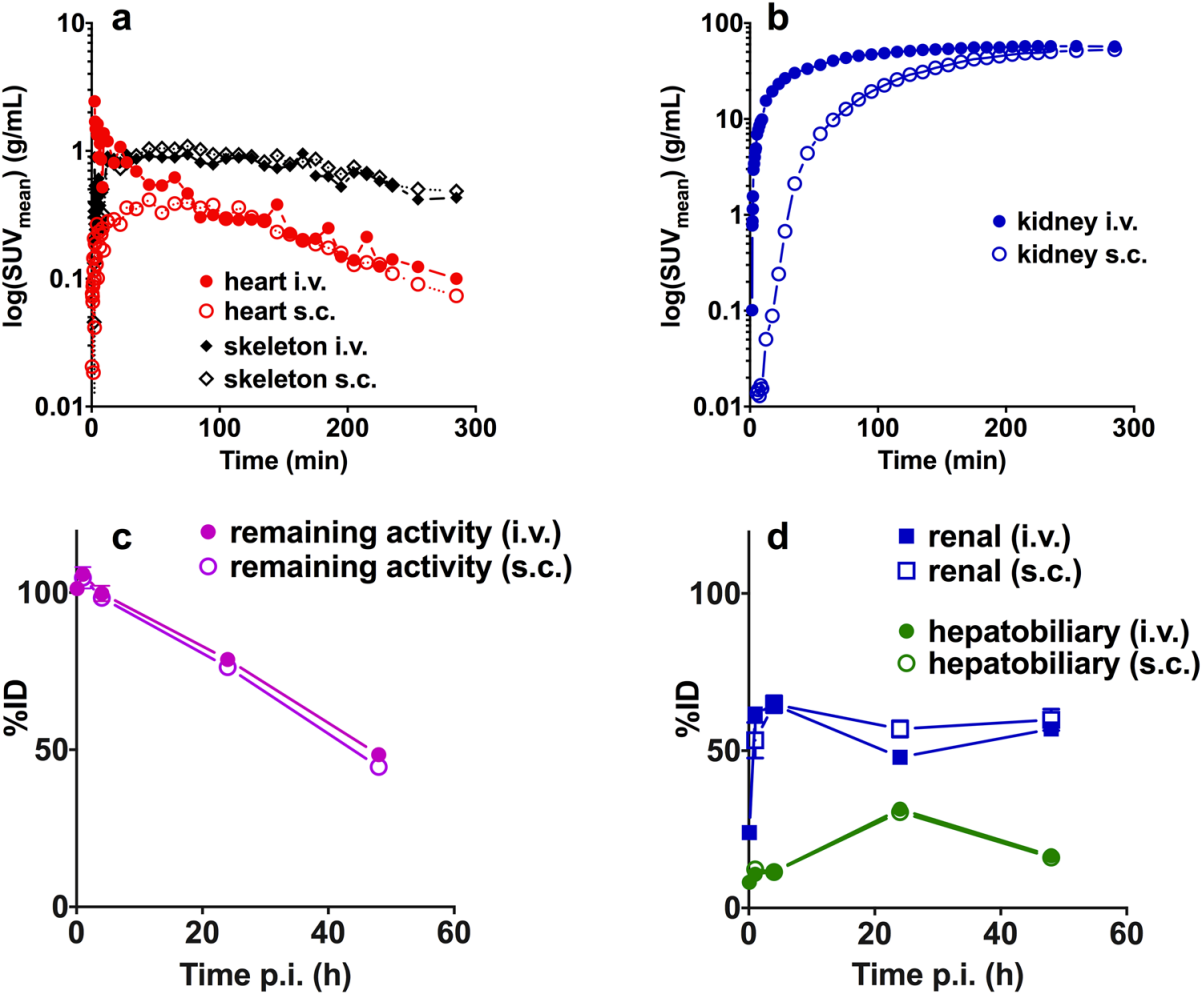
Kinetics in the heart, skeleton, kidneys and elimination. Representative time-activity curves of the [^64^Cu]Cu-NOTA-HsTX1[R14A] in PET studies (**a**) heart, skeleton and (**b**) kidneys of rats after single i.v. (n = 1) or subcutaneous s.c. injection (n = 1). Biodistribution data of the (**c**) elimination of the activity from the body after single i.v. (8 animals; mean ± SEM) or s.c. (4 animals; mean ± SEM) injection and (**d**) renal and hepatobiliary elimination of the [^64^Cu]Cu-NOTA-HsTX1[R14A] after single i.v. or s.c. injection.

The time course of the [^64^Cu]Cu-NOTA-HsTX1[R14A] is shown in Fig. 7a and b. The PET shows that after 5 h the activity concentration in the heart, skeleton, and kidneys was the same for both injection sites.

The remaining activity in the body (Fig. 7c) decreased to 48 ± 3%ID (i.v.) and 45 ± 3%ID (s.c.). The biological half-lives of the total [^64^Cu]Cu-NOTA-HsTX1[R14A] activity in the rat body did not differ substantially between the i.v. (45.4 h) and the s.c. (42.0 h) injections.

Elimination (Fig. 7c,d, Supplementary Table S1) was predominantly renal (kidney and urine), with maximum after 4 h of 65 ± 2% ID, while in the same time only 12 ± 3% ID (i.v.) and 11.4 ± 4%ID (s.c.) were found in the liver and intestine. The maximum of the hepatobiliary elimination was observed at one day after injection with 31 ± 3% ID (i.v.) and 30 ± 3% ID (s.c.), although at this time point the renal elimination was also larger, with 48 ± 3% ID (i.v.) and 51 ± 2% ID (s.c.).

### Metabolite analysis

**Figure 8:**
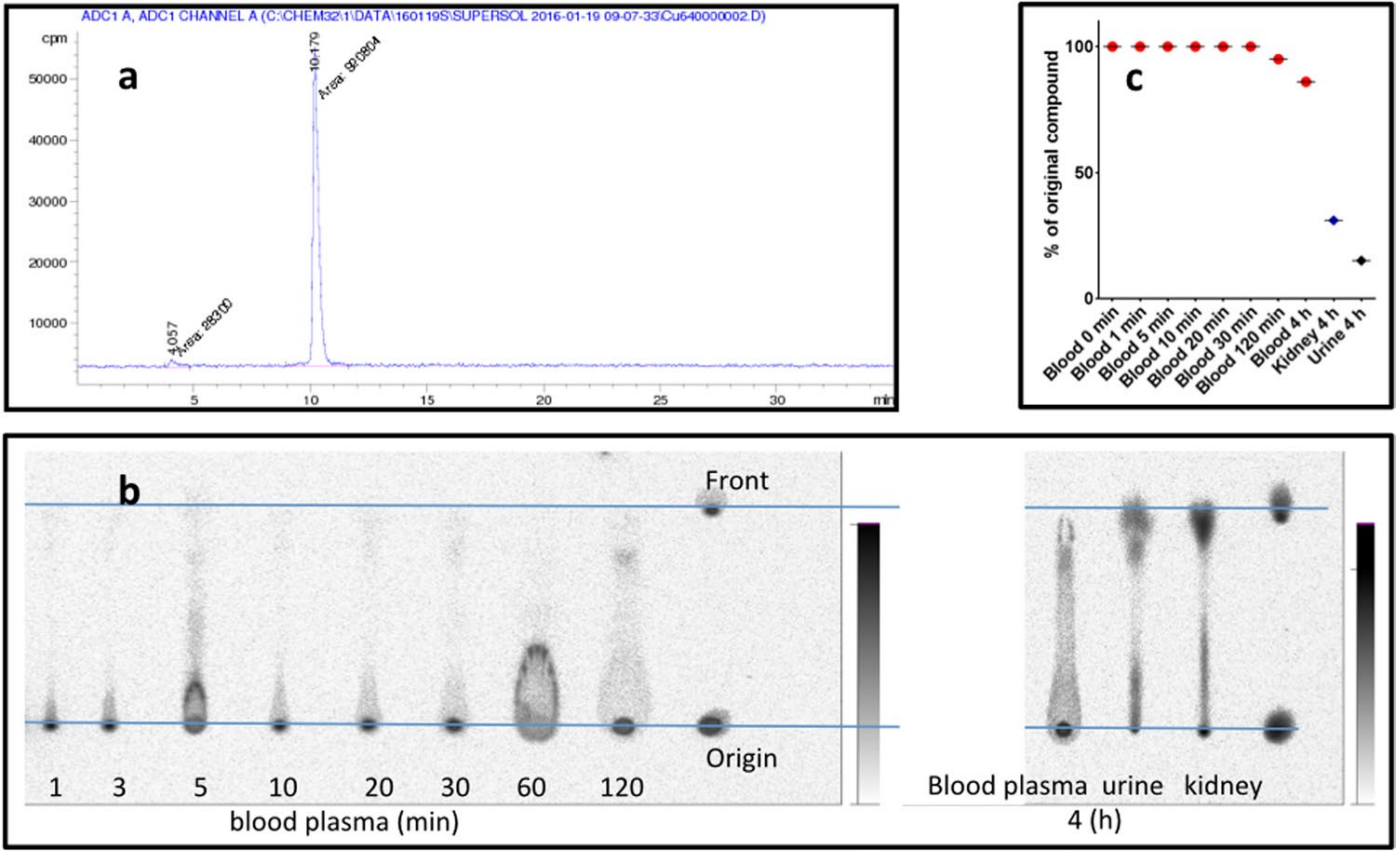
Metabolite analysis. (**a**) Radio-HPLC chromatogram of the [^64^Cu]Cu-NOTA-HsTX1[R14A], (**b**) Radioluminogram of ^64^Cu in a TLC analysis of rat arterial blood plasma samples of 1, 3, 5, 10, 20, 30, 120 min and 4 h, urine and kidney extract samples at 4 h p.i.; (**c**) Radioactive metabolite fraction in the arterial blood plasma samples, kidney extract and urine expressed as % of original compound.

The pharmacokinetics of the [^64^Cu]Cu-NOTA-HsTX1[R14A] includes the distribution, conversion and elimination from the body. The metabolism of [^64^Cu]Cu-NOTA-HsTX1[R14A] was analysed and the data are presented in Fig. 8. To look for potential metabolites, arterial blood samples were taken at different time points and kidney and urine samples were collected after 4 h. The data demonstrate a high metabolic stability of [^64^Cu]Cu-NOTA-HsTX1[R14A] in the arterial blood plasma over the first 2 h without detectable radioactive metabolic products. After 4 h, 85% of the plasma activity was still present as [^64^Cu]Cu-NOTA-HsTX1[R14A]. The largest amounts of activity were accumulated in the kidneys and urine. The analysis showed that the radiotracer was metabolised in the kidneys and excreted into the urine but there was no reuptake into the blood. The ^64^Cu activity in the kidneys and in the urine decreased to 31% and 15% of the original compound during the period of investigation (4 h), respectively. The chemical forms of the radioactive metabolites were not further identified. As a result of the slow renal elimination, blood clearance corrected for the metabolites (Figs 3a and 7a) occurred with a half-life of 37 min (i.v.) and 51 min (s.c.).

## Discussion

HsTX1[R14A] is a potent Kv1.3 channel blocker with excellent selectivity over other potassium channels, and is therefore a potential therapeutic for the treatment of a variety of immune-related diseases associated with chronic inflammation, including systemic or organ-specific autoimmune diseases^13^. An important prerequisite for a therapeutic application is knowledge of its pharmacokinetic and pharmacodynamic properties *in vivo*. ShK-186, for example, was released slowly from the s.c. injection site, resulting in blood concentrations above those required for effective Kv1.3 blockade for up to 7 days in monkeys^8^. As a consequence, single doses of ShK-186 every 2–5 days were as effective as daily administration in delayed-type hypersensitivity, chronic relapsing-remitting experimental autoimmune encephalomyelitis, and pristane-induced arthritis rat models^8^. As the potency of HsTX1[R14A] is similar to that of ShK-186 and the selectivity for Kv1.3 over all other ion channels tested is significantly higher, it was important to assess the biodistribution and elimination of this peptide in animals. We have undertaken these studies in rats as there is a considerable body of evidence indicating that Kv1.3 plays a key role in regulating the membrane potential of T_EM_ cells in this species like in humans^6, 28^.

A ^64^Cu-labelled conjugate of HsTX1[R14A] was used to investigate the pharmacokinetic profile. This peptide appears to be very stable *in vivo*, as anticipated from its structure, which is stabilised by four disulfide bridges, and confirmed experimentally in proteolysis assays^14^. Independent of the route of administration, it was rapidly eliminated from the circulation via the kidneys. The primarily location of the peptide in the arterial blood was the plasma, and there were no indications of significant plasma protein binding. The uptake in erythrocytes did not exceed the normal water space distribution in the red blood cells.

The relatively high accumulation in the epiphysis could be interpreted as binding to activated chondrocyte subpopulations^29^ in the growth plate, a specialised bone marrow cell fraction. Postnatal growth of the long bones occurs through the stimulation of chondrocyte proliferation at the epiphyseal growth plates. The epiphyseal activity is regulated by the growth hormone and other endocrinal factors including insulin-like growth factor 1 (IGF-1). IGF-1-induced proliferation of cells was inhibited by both potassium channel blockers and inhibitors of PI3-kinase. IGF-1 through PI3-kinase, PDK1 and SGK1 up-regulates Kv channels, an effect required for the proliferative action of the growth factor^30, 31^. This could be a mechanism of increased accumulation of the [^64^Cu]Cu-NOTA-HsTX1[R14A] in the bones. However, further experiments are required to substantiate this hypothesis.

The [^64^Cu]Cu-NOTA-HsTX1[R14A] did not show toxic effects in any of the experiments with injections of up to 0.1 mg peptide/kg body weight, even in the anesthetised animals.

The highly stabile, hydrophilic, radiolabelled peptide [^64^Cu]Cu-NOTA-HsTX1[R14A] showed a general biodistribution pattern that is typical for small, metabolically inert pharmaceuticals. The blood clearance was relatively slow, caused by filtration in the kidneys glomeruli. However, the peptide was also trapped in the kidney cortex. The hydrophilic character of the molecule was also reflected in its negligible brain uptake, which makes neurological effects improbable. The low protein binding, minor uptake by macrophages of the lungs or liver and insignificant binding to any scavenger receptors in the blood vessel surface are prerequisites for a high bioavailability of the peptide. Moreover, the selectivity and the low normal-tissue uptake of [^64^Cu]Cu-NOTA-HsTX1[R14A] provide the potential to image regions with increased potassium channel expression.

## Conclusions

The high stability and bioavailability, low normal-tissue uptake of [^64^Cu]Cu-NOTA-HsTX1[R14A], and accumulation in regions of up-regulated Kv channels both *in vitro* and *in vivo* studies demonstrate that the HsTX1[R14A] is a valuable lead compound for the treatment of a variety of immune-related diseases that respond to blockade of the voltage-gated potassium channel Kv1.3. The pharmacokinetics shows that both intravenous and subcutaneous applications are viable routes for the delivery of this potent peptide.

## Methods

### Synthesis of NOTA-functionalised HsTX1[R14A]

The primary assembly of HsTX1[R14A] was completed on a Protein Technologies Prelude synthesizer using an Fmoc-tBu strategy. All eight Cys residues were side-chain protected with the Trt group. All couplings were mediated with diisopropyl carbodiimide/HOBT. Upon completion of the primary chain, an additional AeeA linker was coupled to the N-terminus and subsequently the final Fmoc group was removed and the NOTA(tBu) was coupled as an HOBT-ester. This coupling was monitored via the Kaiser test and judged to be complete after overnight coupling. The peptide was simultaneously cleaved from the resin and deprotected using a TFA-based acidolytic cleavage cocktail with cationic scavengers. The crude peptide was isolated by precipitation in ice-cold ether following filtration through a fritted glass funnel. The crude peptide was subsequently dissolved in 50% aqueous acetic acid and diluted into H_2_O containing 0.1 mM reduced and oxidised glutathione. The pH was adjusted to 7.8 and the solution was slowly stirred for 18 h. The folded peptide was purified by preparative RP-HPLC using a gradient of 5–25% acetonitrile into H_2_O buffered with 0.05% TFA over 90 min. Fractions of the folded peptide were analysed by analytical RP-HPLC and ESI-MS (Supplementary Fig. S1). Fractions with a purity of >96% with the correct mass were pooled together and lyophilised. Yield from the 0.05 mmol synthesis was nearly 30%.

### Electrophysiology

L929 mouse fibroblast cells stably expressing mKv1.3^32^ were gifts from Dr. K. George Chandy (University of California, Irvine). They were maintained in DMEM medium (Invitrogen, Carlsbad, CA) supplemented with 100 IU/mL penicillin, 0.1 µg/mL streptomycin, 2 mM L-glutamine, 10% heat-inactivated foetal bovine serum, and 0.5 mg/mL G418 (EMD Chemicals, Gibbstown, NJ).

Electrophysiology experiments were conducted at room temperature in the whole-cell configuration of the patch-clamp technique on a Port-a-Patch setup (Nanion, North Brunswick, NJ) linked to a HEKA EPC10USB amplifier (HEKA Instruments, Bellmore, NY). A holding potential of −80 mV was used for all recordings. Pipette resistances averaged 2.0 MΩ when filled with internal solution containing (in mM): 145 KF, 10 HEPES, 10 EGTA, and 2 MgCl_2_, pH 7.2, 290 mOsm. Kv currents were elicited every 30 s by 200-ms depolarising pulses from a holding potential of −80 mV to 40 mV. Kv1.3 currents were recorded in normal Ringer solution containing (in mM): 160 NaCl, 4.5 KCl, 2 CaCl_2_, 1 MgCl_2_, and 10 HEPES, pH 7.2, 300 mOsm. IC_50_ values and Hill coefficients were determined by fitting the Hill equation to the reduction of peak current by the drug at 40 mV.

### Radiolabelling

All solvents were purchased from commercial sources (Sigma-Aldrich, Fluka, VWR, Fisher Scientific) and used without further purification. A Direct-Q 3 UV water purification system from Millipore (Merck KGaA) was applied to produce ultrapure water with a resistivity of 18.2 MΩ/cm.

The ^64^Cu production was performed at a PET cyclotron. For the ^64^Ni(p,n)^64^Cu nuclear reaction, 15 MeV protons of a Cyclone^®^ 18/9 with a beam current of 12 µA for 150 min were used. The complete separation of ^64^Cu and ^61^Co was confirmed by gamma-ray spectroscopy. Nickel targets were prepared by electrodeposition of enriched ^64^Ni (99.6%) on gold disks at amounts of 95–120 mg. The plated diameter was 7 mm, matching more than 80% intensity of the cyclotron proton beam, as measured by autoradiography of a ^nat^Ni (natural isotopic composition) disk irradiated with 15 MeV protons to induce the ^nat^Ni(p,x)^57^Ni reaction. The yields of the nuclear reaction ^64^Ni(p,n)^64^Cu were 3.6–5.2 GBq [at end of bombardment (EOB)] with specific activities of 150–250 GBq/µmol Cu^33^.

The radiolabelling yield and stability of ^64^Cu-labelled peptide conjugate were monitored with radio-TLC (thin layer chromatography). Thin layer chromatograms were scanned by using a thin layer analyser (Rita Star, Raytest, Germany). The purity of the radiolabelled peptide was assayed using analytical RP-HPLC. Radio-HPLC was performed on a Knauer Smartline System fitted with an activity detector (Ramona Star, Raytest, Germany) using an Aqua C18 column (Phenomenex, 4.6 mm × 250 mm, 5 µm, 125 Å) with a gradient eluent of H_2_O + 0.05% TFA (A) and acetonitrile + 0.05% TFA (B); in 20 min 0–60% of B, 10 min 60–95% of B, 1 mL/min.

^64^Cu-NOTA-HsTX1[R14A] was prepared by incubating ~1.2–24 nmol (5–100 µg) of NOTA-HsTX1[R14A]/100 µL (1 mg/mL H_2_O stock solution) with ^64^CuCl_2_ (~35–55 MBq) dissolved in 0.2 mL of 0.1 M 2-(*N*-morpholino)ethansulfonic acid (MES)/NaOH buffer (pH 6.0) at 37 °C for 30 min. Formation of the ^64^Cu^II^ complex was verified by radio-TLC on iTLC-SA plates (instant TLC medium impregnated with salicylic acid, Agilent Technology) using H_2_O + 0.1% HCOOH as mobile phase (^64^Cu(HCOO)_2_, *R*_f_ = 0.4, ^64^Cu-NOTA-peptide, *R*_f_ = 0). The stability of the ^64^Cu^II^ formed was assessed in the presence of 0.1 M EDTA solution. Radio-TLC was performed with iTLC-SA plates (Agilent Technology) using 0.1 M aqueous EDTA solution [(^64^Cu]Cu-NOTA-peptide, *R*_f = _0, ^64^Cu-EDTA, *R*_f_ = 0.9).

For *in vivo* experiments, 10 µg NOTA-HsTX1[R14A] in 100 µL buffer was labelled with [^64^Cu]CuCl_2_ (50 MBq) yielding a molar activity of about 20 GBq/µmol. 0.3 MBq aliquots in 500 µL electrolyte solution were injected.

### Animal experiments

Animal experiments were carried out according to the guidelines of the German Regulations for Animal Welfare. The local Ethical Committee for Animal Experiments approved the animal facilities and the protocol according to institutional guidelines and the German animal welfare regulations (reference numbers 24D-9168.11–4/2007-2 and 24-9168.21-4/2004-1). Male Wistar rats (Harlan Winkelmann GmbH, Borchen, Germany) between 7 and 9 weeks of age were housed in an Animal Biosafety Level 1 (ABSL-1) acclimatised facility with a temperature of 22 ± 2 °C and humidity of 55 ± 5%. Animals were kept under a 12 h light cycle in temperature-controlled airflow cabinets (27 ± 1 °C) and had free access to standard pellet feed and water.

### *In vivo* biodistribution

The present study includes data obtained from a total of 50 animals. Four animals (body weight 227 ± 10 g) for each time point were injected intravenously into a tail vein or subcutaneously with approximately 0.3 MBq (0.8 µCi) [^64^Cu]Cu-NOTA-HsTX1[R14A] conjugate in 0.5 mL electrolyte solution E-153 (Serumwerk Bernburg, Germany). Animals were euthanised at 5, 60, 240, 1440 and 2880 min post-injection. Blood and the major organs were collected, weighed, and counted in a cross-calibrated γ-counter (Isomed 1000, Isomed GmbH, Dresden) and Wallac WIZARD Automatic Gamma Counter (PerkinElmer, Germany). The radioactivity of the tissue samples was decay-corrected and calibrated by comparing the counts in tissue with the counts in aliquots of the injected radiotracer that had been measured in the γ-counter at the same time. The activity in the selected organs was expressed as percent-injected dose per organ (% ID) and the activity concentration in tissues and organs as standardised uptake value (SUV). Values are quoted as mean ± standard error of mean (SEM) for each group of four animals.

### Radioluminography

Radioluminography was performed on fresh-frozen sections of 2 thighs from one animal frozen in 2-methylbutane (cooled to −25 °C with liquid nitrogen) and cut with a Leica-CM-1850 cryotom (Leica, Germany) into 10 µm-thick sections. Selected sections were mounted onto microscope slides, air-dried and exposed to a high-performance, high-resolution, water-resistant storage phosphor screen (BAS SR 2025, Fuji Photo Film Co. Ltd., Japan). The exposed imaging plates were scanned in a FUJI BAS 5000 device (Fuji Photo Film Co. Ltd., Japan). The image data were recorded and expressed as digital photo-stimulated luminescence (PSL). Analysis was performed with the AIDA 2.11 program (Raytest, Germany). The whole body cryostat sections were scanned for histologic-autoradiography comparison.

### PET scans

The procedures are described in detail elsewhere^24, 34, 35^. Rats were anesthetised using 9% ± 1% desflurane in 30% oxygen and placed on a heat mat. The animals were kept warm under anesthesia until the end of the scan with a total duration of 4 h. The anesthetised animals were localised in a prone position in the axial direction of the scanner. A needle catheter was installed in a lateral tail vein or subcutaneously in the neck for injection using a syringe pump. PET studies were performed with the dedicated small animal PETs NanoPET/CT (Mediso, Budapest, Hungary) and microPET^(R)^ P4 (Siemens Medical Solutions, Erlangen, Germany). Transmission correction was performed with computer tomography attenuation or transmission scans of 10 min using a ^57^Co point source that were performed before tracer application. Data were acquired over 300 min. Simultaneous with the start of data acquisition, was the infusion of approximately 20 MBq [^64^Cu]Cu-NOTA-HsTX1[R14A] in saline with a duration of 5 min initialised. The PET images were iteratively reconstructed by a 3-dimensional ordered-subset expectation maximisation algorithm (3D OSEM/MAP) with transmission correction and with voxel size of 0.050 × 0.050 × 0.050 cm. No additional corrections were made of partial-volume effects and recovery. Three-dimensional regions of interest (ROI) were determined for subsequent data analysis. The standardised uptake values (SUV, g/mL) and standardised uptake ratios (SUR, as ratio of the SUVs of the tissue of interest and the blood SUV, derived from a region over the caudal arteria abdominalis and vena cava) were used to quantify the activity uptake and kinetics.

### Metabolite analysis

The analysis of [^64^Cu]Cu-NOTA-HsTX1[R14A] degradation was analysed in one rat after single intravenous injection. The gas-anaesthetised (8% desflurane, 30% oxygen, air) rat was prepared on a heating bed for the blood sampling. A needle catheter was inserted in a tail vein and fixed for the injection of the radiotracer and supplementation of the withdrawn volume by E-153 (electrolyte infusion solution). A small incision on the right hind limb was made and a small superficial artery was exposed. A thin tube (polyethylene, 0.3 mm inner and 0.6 mm outer diameter) was inserted, moved to the femoral artery and fixed. Arterial blood samples were collected at 1, 3, 5, 10, 20, 30, 120 min p.i of the [^64^Cu]Cu-NOTA-HsTX1[R14A]. After the end of experiment the anaesthetised animals were euthanised by infusion of KCl solution. For the differentiation of the activity distribution in the blood compartments (Fig. 3b) were blood samples centrifuged (3 min at 14,000 × g) to obtain the plasma (plasma total and blood cells in the precipitate). One kidney was homogenised with PBS (30% w/v) and then centrifuged as described for the blood samples. Urine was treated in analogy. All the supernatants from blood, kidney and urine samples were mixed with equal volume of MeCN to precipitate the protein fraction. The samples were again centrifuged (3 min at 14,000 × g), resulting in the precipitate the protein bound fraction (plasma proteins) and the supernatants (*e.g*. plasma water fraction) were analysed by radio-TLC with RP18 aluminium foil, MeCN/0.1% TFA mixed with H_2_O/0.1% TFA (v/v 1:1). The TLC plates were measured by radioluminography as described above. Aliquots of the whole blood samples and of all supernatants were measured for the ^64^Cu activity concentration in a well counter.

### Statistical analysis

Statistical analyses were carried out with GraphPad Prism version 6 (GraphPad Software, San Diego California USA, www.graphpad.com). The data are expressed as mean ± SD or SEM when indicated. The unmatched biodistribution data at different time points after injection are expressed as % ID (activity amounts) and SUV (activity concentration) were submitted to one-way analysis of variance (ANOVA) followed by Tukeys post-hoc analysis for all data sets to compare the effect of the i.v. and s.c. injection on the means between the groups. Values of p < 0.05 were considered statistically significant and indicated by an asterisk (*), p < 0.01 (**), p < 0.001 (***), p < 0.0001 (****).

## Electronic supplementary material

Supplementary Information
