# Supplementary material for: Distribution and kinetics of the Kv1.3-blocking peptide HsTX1[R14A] in experimental rats
Source: Sci Rep. 2017 Jun 16;7:3756. doi: 10.1038/s41598-017-03998-x (PMC5473807; doi:10.1038/s41598-017-03998-x)
Supplement: Supplementary file 1 — Supplementary Information [file 41598_2017_3998_MOESM1_ESM.pdf]

SUPPLEMENTARY INFORMATION

Distribution and kinetics of the Kv1.3-blocking peptide HsTX1[R14A] in experimental rats

Ralf Bergmann[1], Manja Kubeil[1,2], Kristof Zarschler[1], Sandeep Chhabra[3], Rajeev B. Tajhya[4], Christine Beeton[4], Michael W. Pennington[5], Michael Bachmann[1], Raymond S. Norton[3], Holger Stephan[1]

[1] *Helmholtz-Zentrum Dresden - Rossendorf, Institute of Radiopharmaceutical Cancer Research, Dresden, D-01328, Germany*

[2] *School of Chemistry, Monash University, Melbourne, Victoria 3800 Australia*

[3] *Medicinal Chemistry, Monash Institute of Pharmaceutical Sciences, Monash University, Parkville, Victoria 3052 Australia*

[4] *Department of Molecular Physiology and Biophysics, Baylor College of Medicine, Houston, TX 77030, U.S.A.*

[5] *Peptides International, Louisville, KY 40299, U.S.A.*

Correspondence and requests for materials should be addressed to H.S. (h.stephan@hzdr.de) or R.S.N. (ray.norton@monash.edu)

Supplementary Figures

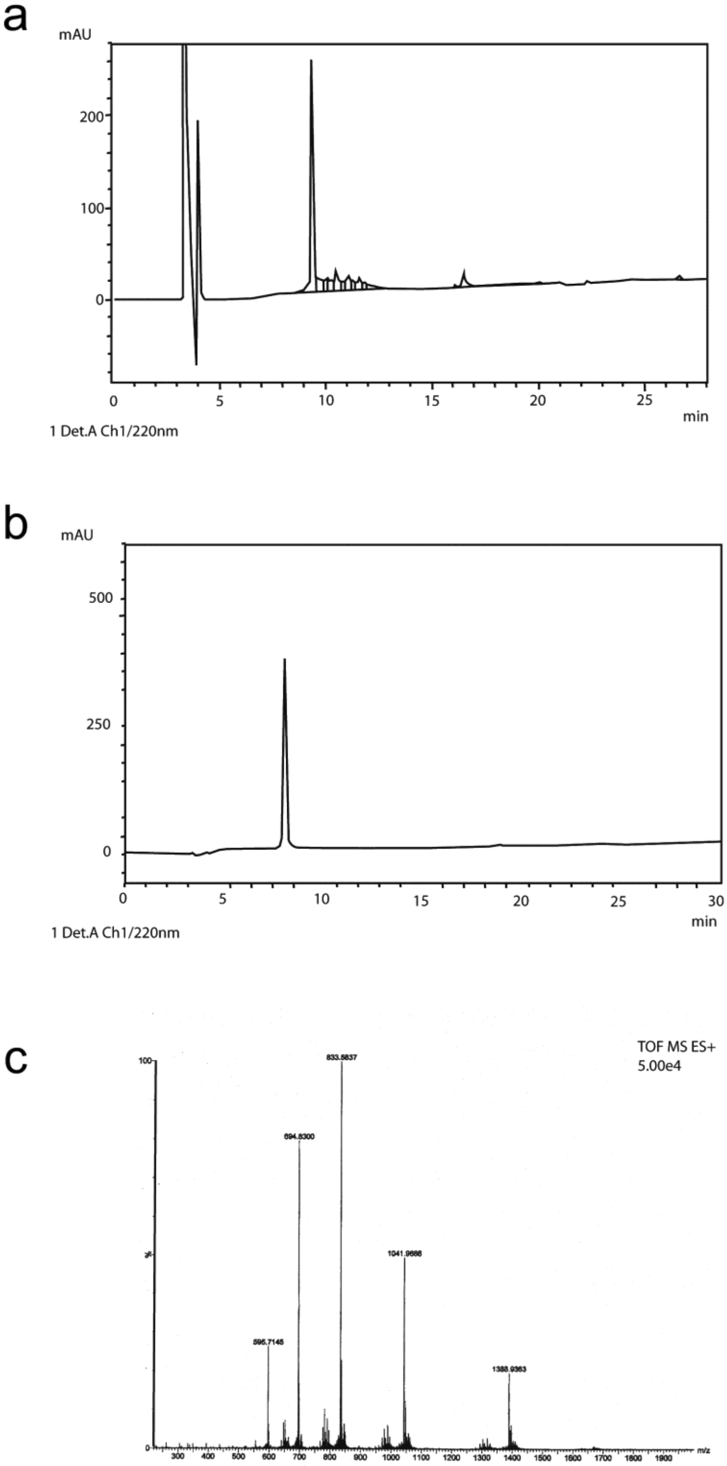

Figure S1. (a) Crude HPLC profile of NOTA-HsTX1[R14A] after 18 h folding according to the Materials and Methods. Gradient conditions are 10-70% B over 30 min at 1 mL/min. Absorbance measured at 220 nm. (b) Purified NOTA-HsTX1[R14A] using gradient of 5-45% B over 30 min. (c) ESI-MS demonstrating multi-charged ions for the purified, oxidised NOTA-HsTX1[R14A].

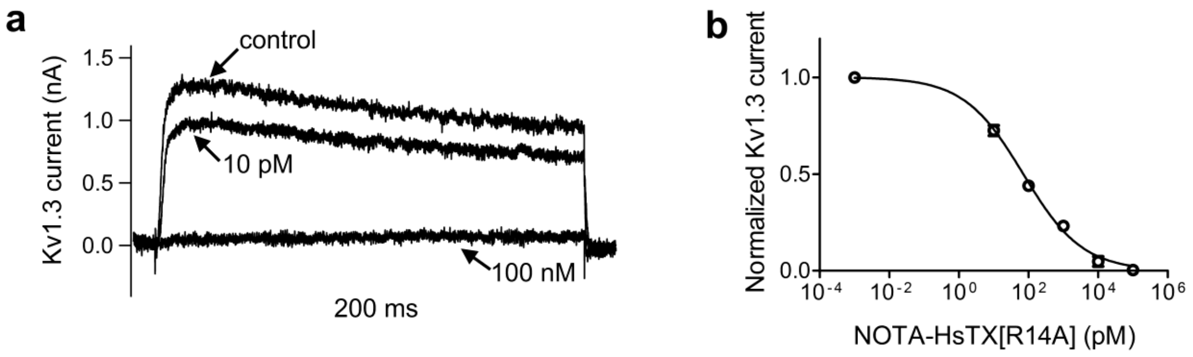

Figure S2. Functional analyses of NOTA-HsTX1[R14A]. (a) Whole-cell Kv1.3 currents measured by whole-cell patch-clamp in stably transfected L929 fibroblasts before (control) and after perfusion of 10 pM and 100 nM NOTA-HsTX1[R14A]. (b) Dose-response inhibition of Kv1.3 current by NOTA-HsTX1[R14A] fitted to a Hill equation (n = 3 cells per concentration).

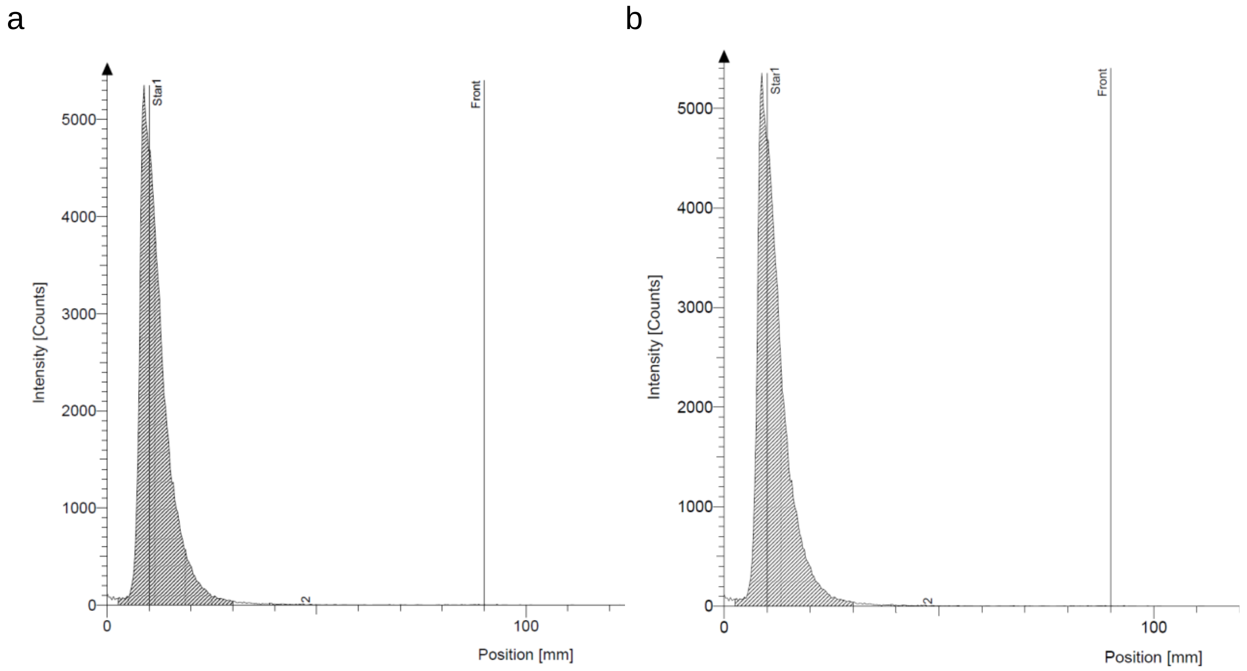

Figure S3. Radio-TLC chromatograms of [^{64}Cu]Cu-NOTA-HsTX1[R14A] conjugate ($R_f = 0$), RCY of conjugate > 99%; (a) iTLC-SA, 0.1 M aq. EDTA solution, (b) iTLC-SA, 0.1% aq. HCOOH.

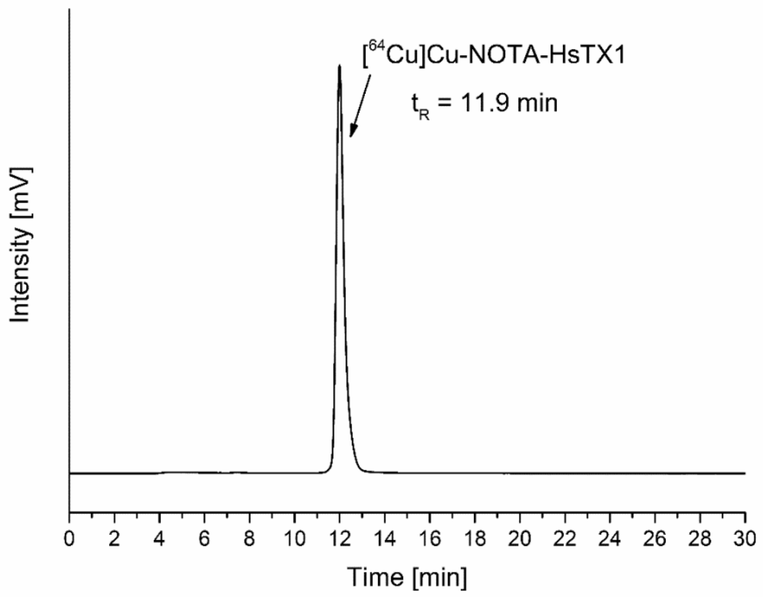

Figure S4. Radio-HPLC chromatogram of [^{64}Cu]Cu-NOTA-HsTX1[R14A], RCP > 99% (free $^{64}Cu^{II}$, t_R ~ 4 min); Aqua C18 column (Phenomenex, 4.6 mm × 250 mm, 5 µm, 125 Å) using a gradient eluent of H_2O + 0.05% TFA (A) and CH_3CN + 0.05% TFA (B); in 20 min 60% of B, in 10 min 95% B, 1 mL/min.

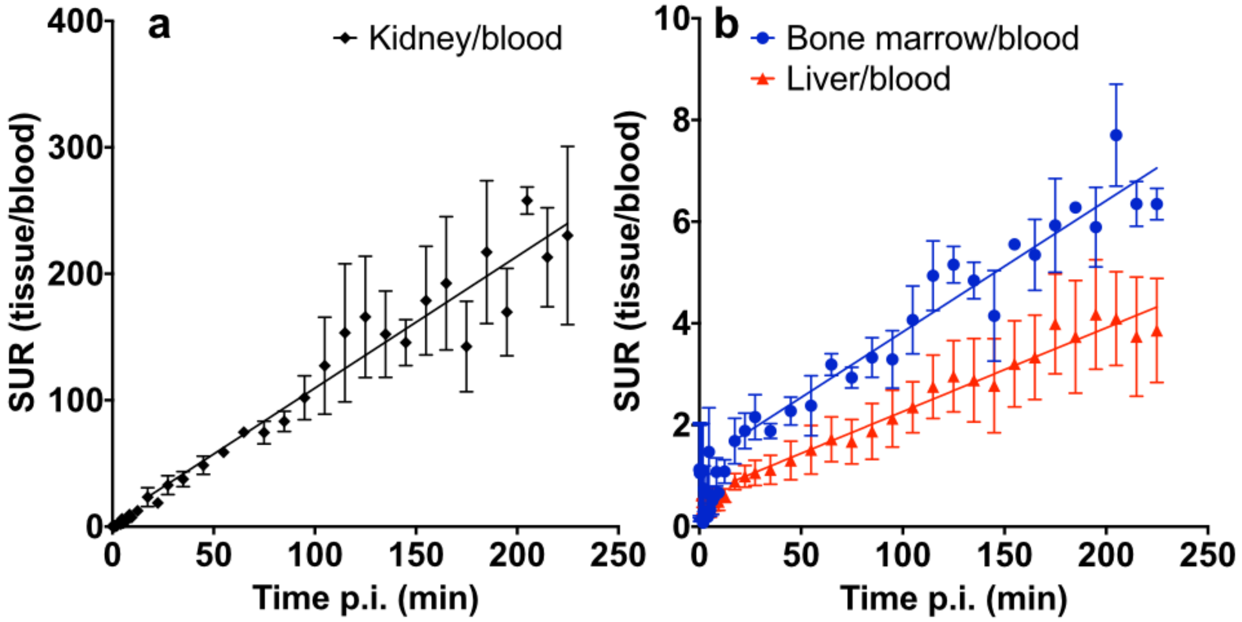

Figure S5. Standard uptake ratios (tissue to blood ratios) of (a) kidney, (b) bone marrow and liver; calculated from the PET studies after i.v. injection (mean ± SEM, n = 3).

Table S1. Biodistribution of [^{64}Cu]Cu-NOTA-HsTX1[R14A] in male Wistar rats after single intravenous and subcutaneous injection. Activity amounts are expressed as percent of injected dose (%ID) as mean ± SD (number of animals).

**%ID**			i.v.				s.c.		
Time p.i. (min)	5	60	240	1440	2880	60	240	1440	2880
Brain	0.05 ± 0.00 (4)	0.05 ± 0.03 (8)	0.05 ± 0.01 (4)	0.08 ± 0.03 (8)	0.10 ± 0.00 (4)	0.06 ± 0.01 (4)	0.05 ± 0.00 (4)	0.10 ± 0.00 (4)	0.09 ± 0.00 (4)
Pancreas	0.11 ± 0.03 (4)	0.06 ± 0.02 (8)	0.08 ± 0.00 (4)	0.20 ± 0.16 (8)	0.08 ± 0.01 (4)	0.11 ± 0.03 (4) *	0.08 ± 0.01 (4)	0.15 ± 0.01 (4)	0.07 ± 0.01 (4)
Spleen	0.83 ± 0.12 (4)	0.79 ± 0.11 (8)	0.67 ± 0.06 (4)	0.18 ± 0.04 (8)	0.16 ± 0.01 (4)	1.08 ± 0.03 (4) **	0.75 ± 0.03 (4)	0.15 ± 0.00 (4)	0.15 ± 0.01 (4)
Adrenals	0.03 ± 0.00 (4)	0.01 ± 0.00 (8)	0.01 ± 0.00 (4)	0.02 ± 0.00 (8)	0.02 ± 0.00 (4)	0.01 ± 0.00 (4)	0.01 ± 0.00 (4)	0.02 ± 0.00 (4)	0.02 ± 0.01 (4)
Kidneys	23.9 ± 3.93 (4)	61.6 ± 4.21 (8)	63.1 ± 0.97 (4)	14.2 ± 2.04 (8)	6.19 ± 0.49 (4)	53.3 ± 4.90 (4) *	62.5 ± 2.36 (4)	13.6 ± 1.10 (4)	4.95 ± 0.49 (4)
Heart	0.33 ± 0.04 (4)	0.21 ± 0.06 (8)	0.19 ± 0.03 (4)	0.23 ± 0.02 (8)	0.27 ± 0.04 (4)	0.32 ± 0.02 (4) *	0.18 ± 0.03 (4)	0.23 ± 0.01 (4)	0.22 ± 0.01 (4)
Lung	1.44 ± 0.18 (4)	1.24 ± 0.30 (8)	0.92 ± 0.12 (4)	0.50 ± 0.05 (8)	0.46 ± 0.06 (4)	1.64 ± 0.24 (4)	1.04 ± 0.08 (4)	0.49 ± 0.02 (4)	0.33 ± 0.01 (4)
Thymus	0.37 ± 0.07 (4)	0.37 ± 0.08 (8)	0.37 ± 0.04 (4)	0.21 ± 0.03 (8)	0.17 ± 0.02 (4)	0.46 ± 0.01 (4)	0.43 ± 0.04 (4)	0.22 ± 0.01 (4)	0.16 ± 0.02 (4)
Thyroid	0.07 ± 0.00 (4)	0.11 ± 0.02 (8)	0.12 ± 0.01 (4)	0.08 ± 0.01 (8)	0.07 ± 0.02 (4)	0.13 ± 0.02 (4)	0.11 ± 0.02 (4)	0.09 ± 0.05 (4)	0.03 ± 0.02 (4)
Harderian gl.	0.10 ± 0.02 (4)	0.04 ± 0.00 (8)	0.03 ± 0.00 (4)	0.08 ± 0.01 (8)	0.07 ± 0.00 (4)	0.04 ± 0.00 (4)	0.03 ± 0.00 (4)	0.08 ± 0.01 (4)	0.06 ± 0.01 (4)
Liver	4.60 ± 2.22 (4)	5.43 ± 1.44 (8)	7.01 ± 1.47 (4)	14.8 ± 2.42 (8)	8.35 ± 0.95 (4)	4.44 ± 0.74 (4)	6.79 ± 2.27 (4)	11.1 ± 1.82 (4)	7.85 ± 0.49 (4)
Femur	1.32 ± 0.89 (4)	0.80 ± 0.21 (8)	0.55 ± 0.03 (4)	0.30 ± 0.06 (8)	0.23 ± 0.01 (4)	0.82 ± 0.04 (4)	0.64 ± 0.02 (4) *	0.34 ± 0.01 (4)	0.19 ± 0.02 (4)
Testes	0.27 ± 0.05 (4)	0.23 ± 0.02 (8)	0.21 ± 0.01 (4)	1.05 ± 0.13 (8)	0.76 ± 0.02 (4)	0.21 ± 0.01 (4)	0.20 ± 0.03 (4)	0.92 ± 0.06 (4)	0.63 ± 0.06 (4)
Intestine	3.59 ± 2.40 (4)	5.30 ± 2.31 (8)	4.61 ± 1.84 (4)	16.5 ± 2.32 (8)	8.27 ± 1.01 (4)	7.85 ± 3.82 (4)	4.65 ± 1.73 (4)	19.3 ± 1.35 (4)	8.16 ± 1.02 (4)
Stomach	0.67 ± 0.09 (4)	1.28 ± 1.20 (8)	1.52 ± 1.06 (4)	1.56 ± 1.04 (8)	1.46 ± 0.37 (4)	1.14 ± 0.45 (4)	0.60 ± 0.03 (4)	0.82 ± 0.07 (4)	1.72 ± 1.02 (4)
Carcass	63.4 ± 6.19 (4)	28.1 ± 1.55 (8)	20.1 ± 1.68 (4)	28.4 ± 3.49 (8)	21.6 ± 1.04 (4)	32.9 ± 2.62 (4) **	20.1 ± 1.28 (4)	28.5 ± 1.49 (4)	19.7 ± 2.74 (4)

Table S2. Biodistribution of [^{64}Cu]Cu-NOTA-HsTX1[R14A] in male Wistar rats after single intravenous and subcutaneous injection. Activity concentrations in the tissues are expressed as activity per g tissue normalized to the injected activity and body weight in g, standard uptake value (SUV, g/g) as mean ± SD (number of animals)..

**SUV**	i.v.					s.c.			
Time p.i. (min)	5	60	240	1440	2880	60	240	1440	2880
Blood	1.09 ± 0.62 (4)	0.70 ± 0.23 (8)	0.51 ± 0.02 (4)	0.71 ± 0.16 (8)	0.38 ± 0.02 (4)	0.65 ± 0.11 (4)	0.47 ± 0.21 (4)	0.44 ± 0.04 (4)	0.30 ± 0.01 (4)
Brain	0.07 ± 0.00 (4)	0.05 ± 0.01 (8)	0.03 ± 0.00 (4)	0.08 ± 0.01 (8)	0.07 ± 0.00 (4)	0.04 ± 0.00 (4)	0.03 ± 0.00 (4)	0.06 ± 0.00 (4)	0.07 ± 0.00 (4)
Pancreas	0.64 ± 0.09 (4)	0.30 ± 0.04 (8)	0.24 ± 0.01 (4)	0.62 ± 0.16 (8)	0.32 ± 0.01 (4)	0.29 ± 0.03 (4)	0.25 ± 0.00 (4)	0.48 ± 0.02 (4)	0.28 ± 0.02 (4)
Spleen	4.06 ± 0.60 (4)	3.42 ± 0.42 (8)	2.40 ± 0.10 (4)	0.83 ± 0.26 (8)	0.56 ± 0.05 (4)	4.00 ± 0.21 (4) *	2.84 ± 0.25 (4) *	0.59 ± 0.03 (4)	0.47 ± 0.04 (4)
Adrenals	1.26 ± 0.48 (4)	0.65 ± 0.37 (8)	0.39 ± 0.05 (4)	0.86 ± 0.11 (8)	0.71 ± 0.04 (4)	0.40 ± 0.08 (4)	0.40 ± 0.02 (4)	0.73 ± 0.10 (4)	0.55 ± 0.33 (4)
Kidneys	35.1 ± 6.82 (4)	78.4 ± 13.6 (8)	68.4 ± 4.28 (4)	18.0 ± 5.32 (8)	6.37 ± 0.59 (4)	58.1 ± 5.55 (4) *	70.2 ± 4.14 (4)	14.4 ± 1.32 (4)	5.98 ± 1.08 (4)
WAT	0.28 ± 0.06 (4)	0.19 ± 0.07 (8)	0.15 ± 0.00 (4)	0.19 ± 0.09 (8)	0.34 ± 0.08 (4)	0.25 ± 0.05 (4)	0.17 ± 0.05 (4)	0.22 ± 0.01 (4)	0.17 ± 0.08 (4)
Muscle	0.43 ± 0.03 (4)	0.17 ± 0.06 (8)	0.07 ± 0.00 (4)	0.18 ± 0.02 (8)	0.19 ± 0.01 (4)	0.15 ± 0.02 (4)	0.08 ± 0.01 (4)	0.19 ± 0.01 (4)	0.16 ± 0.00 (4)
Heart	1.04 ± 0.14 (4)	0.53 ± 0.08 (8)	0.40 ± 0.07 (4)	0.62 ± 0.07 (8)	0.64 ± 0.06 (4)	0.70 ± 0.03 (4) **	0.41 ± 0.06 (4)	0.53 ± 0.02 (4)	0.57 ± 0.04 (4)
Lung	2.87 ± 0.32 (4)	1.98 ± 0.17 (8)	1.24 ± 0.10 (4)	0.78 ± 0.13 (8)	0.55 ± 0.03 (4)	2.10 ± 0.33 (4)	1.32 ± 0.12 (4)	0.63 ± 0.02 (4)	0.47 ± 0.01 (4)
Thymus	1.51 ± 0.08 (4)	1.33 ± 0.19 (8)	1.07 ± 0.02 (4)	0.74 ± 0.15 (8)	0.53 ± 0.02 (4)	1.15 ± 0.03 (4)	1.07 ± 0.11 (4)	0.63 ± 0.05 (4)	0.46 ± 0.05 (4)
Harderian gl.	0.82 ± 0.12 (4)	0.30 ± 0.05 (8)	0.23 ± 0.01 (4)	0.56 ± 0.10 (8)	0.48 ± 0.05 (4)	0.32 ± 0.01 (4)	0.21 ± 0.01 (4)	0.46 ± 0.01 (4)	0.44 ± 0.02 (4)
Liver	1.02 ± 0.53 (4)	1.24 ± 0.31 (8)	1.78 ± 0.45 (4)	2.93 ± 0.78 (8)	1.45 ± 0.13 (4)	1.13 ± 0.23 (4)	1.75 ± 0.63 (4)	2.23 ± 0.32 (4)	1.40 ± 0.08 (4)
Femur	3.04 ± 2.13 (4)	1.58 ± 0.58 (8)	0.87 ± 0.04 (4)	0.54 ± 0.06 (8)	0.39 ± 0.02 (4)	1.25 ± 0.07 (4)	0.92 ± 0.08 (4)	0.47 ± 0.02 (4)	0.34 ± 0.02 (4)
Testes	0.24 ± 0.03 (4)	0.16 ± 0.01 (8)	0.13 ± 0.01 (4)	0.87 ± 0.34 (8)	0.50 ± 0.02 (4)	0.15 ± 0.01 (4)	0.12 ± 0.01 (4)	0.54 ± 0.02 (4)	0.44 ± 0.01 (4)

Table S3. Pharmacokinetic parameters calculated from the biodistribution data.

Parameter		Blood i.v.	Blood s.c.	Liver i.v.	Liver s.c.	Kidney i.v.	Kidney s.c.
**animals**	**n**	8	4	8	4	8	4
**AUC***	**(SUV x h)**	28.3 ± 2,1	19,9 ± 2,6	92,7 ± 11,0	87,8 ± 9,5	1417 ± 102	1285 ± 57
**$t_{1/2}$****	**(h)**	49,5	46,4	39,5	35,9	14,7	18,9
**Peak time**	**(h)**	1	1	24	24	1	4
**Cmax**	**(SUV)**	0,83 ± 0,26	0,65 ± 0,11	2,50 ± 0,57	2,23 ± 0,32	74,8 ± 12,8	70,2 ± 4,1

* AUC was calculated between 1 and 48 h

** $t_{1/2}$ was calculated from 24 to 48 h.
